# COVID-19: Defining an invisible enemy within healthcare and the community

**DOI:** 10.1017/ice.2020.283

**Published:** 2020-06-08

**Authors:** Saif A. Musa, Anand Sivaramakrishnan, Stephanie Paget, Husam El-Mugamar

**Affiliations:** 1 Department of Gastroenterology, Chase Farm Hospital, Royal Free London NHS Foundation Trust, Enfield, Middlesex, England; 2 Department of Microbiology, Barnet Hospital, Royal Free London NHS Foundation Trust, Barnet, England

*To the Editor—*The rapid dissemination of severe respiratory coronavirus virus 2 (SARS-CoV-2) throughout the globe has been declared a pandemic. A lack of national and internationally agreed case definitions for healthcare-associated coronavirus disease 2019 (COVID-19) has led to inconsistencies in describing epidemiology, which limit comparisons.^1,2^ A median incubation period of 5 (range, 1–14) days has been accepted in COVID-19 guidance.^3^ Adapting established case definitions from other infectious diseases, such as *Clostridium difficile* infection (CDI), may help overcome variability.^4^ All cases with a positive nasopharyngeal real-time polymerase chain reaction (PCR) assay would therefore be described as either healthcare associated (HA) or community associated (CA).

Hospital-onset healthcare-associated (HoHA) COVID-19 would define current hospitalized inpatients residing >14 days. Hospital-onset possible healthcare-associated (HoPHA) cases, in those residing between 3 and 14 days in the hospital, in the absence of suspected COVID-19 on admission. New cases diagnosed within 14 days of acute-care hospital discharge would be community-onset, healthcare-associated (CoHA) infection. Community-associated (CA) cases would refer to those diagnosed within 2 days or suspected on admission (diagnosed >2 days after admission) and no acute-care hospitalization within the previous 14 days. This group can be further subdivided into those who are independent and self-caring from their own home (CoCA) or those requiring social care, that is, social-onset community-associated (SoCA). Social care includes those requiring domiciliary care (including visiting home care, extra care housing and live in homecare), admissions from care homes, community rehabilitation, and mental healthcare institutions.

**Fig. 1.**
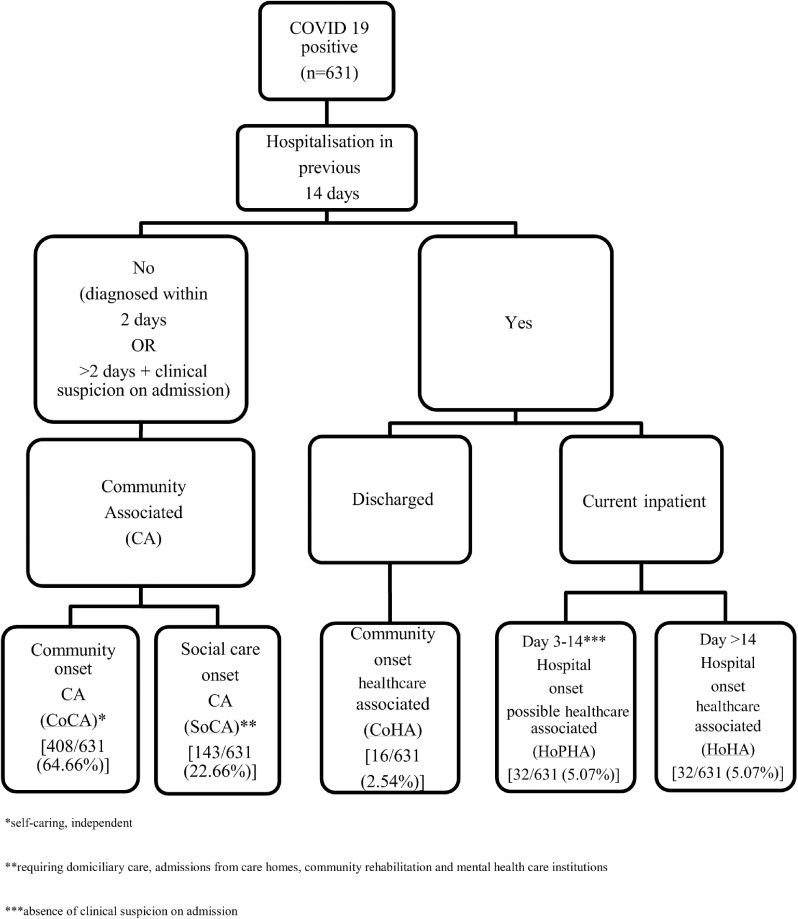
Algorithm describing COVID 19 cases presenting at our institution from March 1, 2020, to April 15, 2020.

We retrospectively applied these definitions to 631 adult COVID-19–positive patients (aged >16 years) at our acute-care institution in North London from March 1, 2020, to April 15, 2020 inclusive. The study was registered with our local clinical governance committee. Because all care was routine, in keeping with UK national guidance, ethical approval was not required. In total, HoHA, HoPHA, and CoHA cases accounted for 80 of 631 (12.68%) of all positive cases (Fig. 1). The rates of HoHA and HoPHA COVID-19 cases per total number of hospital admissions during this period were 32 of 1,818 (1.76%) and 32 of 1,818 (1.76%), respectively. Median diagnosis occurred 25 days (IQR, 21–43) after admission for HoHA COVID-19 and 9 days (IQR, 6–11) after admission for HoPHA COVID-19, respectively. For CoHA, 16 of 631 patients (2.54%) presented a median of 8 days (IQR, 2–11) after hospital discharge. Median diagnosis of CA cases occurred 1 day (IQR, 1–2) after testing.

Recent NHS England guidance recommends screening all emergency hospital admissions on admission followed by a single repeat, for those testing negative, between 5 and 7 days after admission.^5^ Our data demonstrate that healthcare-associated COVID-19 has contributed an important number of cases patients during the height of a pandemic. Sequential screening of non–COVID-19 hospitalized patients beyond this, possibly on a weekly basis up to 14 days after hospital discharge, may prove beneficial in further reducing the threat posed by SARS-CoV-2. Further validation of proposed definitions is required and according to the evolution of CDI definitions, amendments are likely.
